# Enhanced Azo-Dyes Degradation Performance of Fe-Si-B-P Nanoporous Architecture

**DOI:** 10.3390/ma10091001

**Published:** 2017-08-27

**Authors:** Nan Weng, Feng Wang, Fengxiang Qin, Wanying Tang, Zhenhua Dan

**Affiliations:** 1 School of Materials Science and Engineering, Nanjing University of Science and Technology, Nanjing 210094, China; nanweng18@163.com (N.W.); fengwang0908@163.com (F.W.); 2 School of Chemical Engineering, Nanjing University of Science and Technology, Nanjing 210094, China; 3 College of Materials Science and Engineering, Nanjing TECH University, Nanjing 210009, China

**Keywords:** dealloying, nanoporous structure, azo dyes, degradation, amorphous alloys

## Abstract

Nanoporous structures were fabricated from Fe_76_Si_9_B_10_P_5_ amorphous alloy annealed at 773 K by dealloying in 0.05 M H_2_SO_4_ solution, as a result of preferential dissolution of α-Fe grains in form of the micro-coupling cells between α-Fe and cathodic residual phases. Nanoporous Fe-Si-B-P powders exhibit much better degradation performance to methyl orange and direct blue azo dyes compared with gas-atomized Fe_76_Si_9_B_10_P_5_ amorphous powders and commercial Fe powders. The degradation reaction rate constants of nanoporous powders are almost one order higher than those of the amorphous counterpart powders and Fe powders, accompanying with lower activation energies of 19.5 and 26.8 kJ mol^−1^ for the degradation reactions of methyl orange and direct blue azo dyes, respectively. The large surface area of the nanoporous structure, and the existence of metalloids as well as residual amorphous phase with high catalytic activity are responsible for the enhanced azo-dyes degradation performance of the nanoporous Fe-Si-B-P powders.

## 1. Introduction

Azo dyes have been widely used in the printing and dyeing industry. Under the special conditions, azo dyes can be disintegrated into more than 20 kinds of aromatic amines which are harmful to the environment. Zero-valent iron (ZVI), such as iron powders and iron-based amorphous alloys, which are inexpensive, resourceful and non-toxic, has been successfully utilized in wastewater treatment industry [1,2]. ZVI has been reported to be efficient in the reductive decomposition of chlorinated organic compound [3,4], heavy metals [5] and azo dye [6]. Rapid decay of the degradation efficiency of the iron powders occurs due to the ready rusting, which restricts the extensive utilization in degrading azo dyes. The degradation efficiency of iron based amorphous alloys in decomposing azo dyes is much higher than that of the crystalline ZVI due to its amorphous structure and the addition of metalloids [7,8,9]. Moreover, a series of research works have been conducted to improve the reactivity of these zero-valent iron materials, such as by improving the specific surface area of iron powders (nanoscale ZVI (NZVI) [10,11]), doping with additional metals (such as Pd [4,12], Ni [13], and Cu [14]) and so on. The design of the chemical composition of the degradation agents is another important aspect for enhancing decomposition of the azo dye pollutants. For example, the existence of the metalloid elements (Si, B) is expected to improve the degradation activity of Fe-based alloys because of the formation of loose oxide layer with high catalytic activity [8].

Moreover, the microstructural optimization of the degradation agents is helpful for increasing the surface area and the activity. It has been reported that iron based amorphous powders fabricated from ball milling with larger surface area exhibit better degradation performance than their counterpart bulk alloys [9]. As is well known, nanoporous metals have attracted considerable interest in catalytic application because of the high specific surface area [14]. Many approaches have been developed to fabricate porous metals [15,16,17]. Among them, chemical or electro-chemical dealloying method is a simple process for producing 3D bi-continuous nanoporosity. The porous nanostructure is ideal for catalysts, chemical sensors and biofilteration because the unique microstructure with ultra-large surface area of the interconnected nanopore channels and metallic ligaments fulfills unlimited transport of medium molecules and electrons [9,15].

In this research, as-spun Fe_76_Si_9_B_10_P_5_ amorphous alloys were nanocrystallized through annealing at 773 K and dealloyed in 0.05 M H_2_SO_4_ solution to fabricate the nanoporous structure with large surface area, which is expected to be beneficial to improving the degradation performance to azo dyes. Moreover, the existence of metalloids such as Si, B and P may enhance the catalytic activity more. Therefore, iron based nanoporous materials with Si, B and P are expected to be promising catalysts in degrading azo dyes owing to their large surface area and the existence of metalloids. Commercial Fe powder, gas-atomized Fe_76_Si_9_B_10_P_5_ powders and Fe-Si-B-P nanoporous powders served as degradation agents to degrade methyl orange (MO) and direct blue (DB), which are typical pollutants in industrial wastewater. The mechanisms of the formation of the nanoporous structure and enhancement of the degradation performance of Fe-Si-B-P nanoporous powders to azo dyes are discussed.

## 2. Materials and Methods

Fe_76_Si_9_B_10_P_5_ alloy ingots were prepared by induction melting with mixtures of Fe (99.98 mass%), Si (99.998 mass%), B (99.5 mass%) and pre-melted Fe-P (99.9 mass%) in a high purity argon atmosphere. A single-roller melt-spinning method was used to produce the rapidly solidified ribbons with a thickness of about 20 μm and a width of about 5 mm. The melt-spun specimens were nanocrystallized through annealing at 773 K for 600 s. The Fe_76_Si_9_B_10_P_5_ amorphous powders were prepared by gas-atomization process and commercial Fe powders were purchased from JFE steels. Hereafter, Fe_76_Si_9_B_10_P_5_ amorphous powders and commercial Fe powders are denoted as AM powders and FE powders, respectively, and investigated as reference materials. The structures of dealloyed Fe_76_Si_9_B_10_P_5_ alloy, Fe_76_Si_9_B_10_P_5_ amorphous powders and commercial Fe powder were identified by using an X-ray diffraction (XRD, Bruker-AXS D8, Karlsruhe, Germany) with Cu Kα radiation. The electrochemical measurements were conducted in a three-electrode cell using a platinum electrode and a saturated calomel electrode (SCE). The potentiodynamic polarization curves were measured in 0.05 M H_2_SO_4_ and simulated pollutant solutions with a scan rate of 50 mV/min.

Annealed Fe_76_Si_9_B_5_P_10_ alloy was dealloyed by immersing in 0.05 M H_2_SO_4_ solution for 3.6 ks in free corrosion condition. The morphology and microstructure of dealloyed specimens were characterized by a scanning electron microscope (SEM, QUANTA FEG 250, Hillsboro, OR, USA) and a transmission electron microscope (TEM, JEOL 2100F, Tokyo, Japan). The porosity of the dealloyed specimens is estimated by a simple method for determining the porosity based on SEM images using PhotoshopPro software (Adobe, Adobe photoshop CC, San Jose, CA, USA). Energy dispersive X-ray spectrometer(EDS) and X-ray photoelectron spectrometer (XPS, THERMO ESCALAB 250XI, Waltham, MA, USA) with monochromatized Al Kα excitation (hν = 1486.6 eV) were employed to analyze the chemical composition and the elemental valences of the surface before and after degradation of the simulated wastewater. Dealloyed Fe_76_Si_9_B_10_P_5_ ribbons were milled to form powders to make it easy to react with azo dye solution. Hereafter, nanoporous Fe_76_Si_9_B_10_P_5_ powders are denoted as NP powders. MO (C_14_H_14_N_3_SO_3_Na) solution with a concentration of 20 mg L^−1^ and DB (C_32_H_20_N_6_S_4_O_10_Na_4_) solution with a concentration of 50 mg L^−1^ were used as a simulation industrial pollutant. The load of degradation powders was 10 g L^−1^. The degradation behavior of NP powder, AM powders and FE powders was investigated at 303, 318 and 333 K. Scanning UV-vis spectroscopy (THERMO EVOLUTION 220, Waltham, MA, USA) was employed to analyze the degradation efficiency of MO and DB solutions.

## 3. Results and Discussion

### 3.1. Characteristics of NP Powders, AM Powders and FE Powders

**Figure 1:**
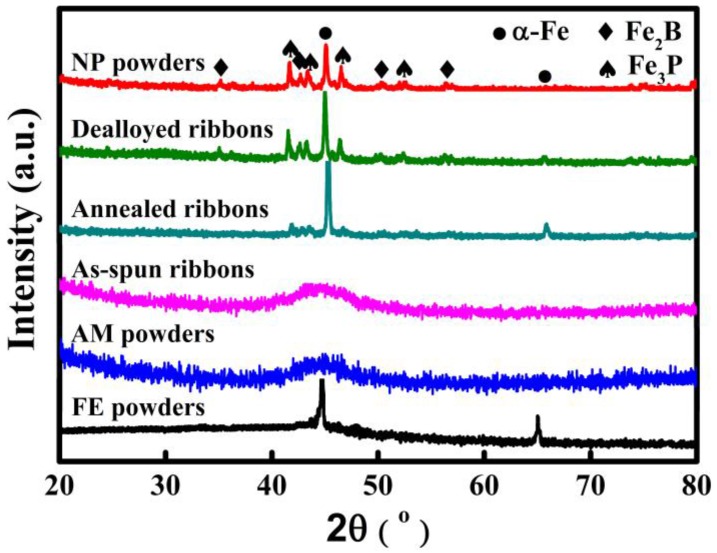
XRD patterns of NP powders, AM powder, FE powders, dealloyed Fe_76_Si_9_B_10_P_5_ ribbons, annealed Fe_76_Si_9_B_10_P_5_ ribbons and as-spun Fe_76_Si_9_B_10_P_5_ amorphous ribbons.

The XRD patterns of NP powders, AM powders and FE powders as well as as-spun ribbons, annealed ribbons and dealloyed ribbons are shown in Figure 1. The appearance of a single halo diffraction peak typical for amorphous alloys indicates that both AM powders and as-spun ribbons are in an amorphous state. The diffraction peaks at about 45.3° and 65.9° of FE powders are assigned to α-Fe phase. Some diffraction peaks originating from α-Fe, Fe_2_B and Fe_3_P are confirmed in the pattern of annealed ribbons, indicating that the α-Fe, Fe_2_B and Fe_3_P phases precipitate after annealing of the as-spun ribbons. The presence of two diffraction peaks at 45.3° and 65.9° from α-Fe crystals in the pattern of NP powders and dealloyed ribbons suggests that some α-Fe phases are residual after dealloying. Meanwhile, the relative diffraction peaks of Fe_2_B and Fe_3_P phases are stronger in the patterns of NP powders and dealloyed ribbons than in as-annealed ribbons. It is thus predicted that α-Fe grains are partially preferentially dissolved in 0.05 M H_2_SO_4_ solution, which may be attributed to the potential difference between α-Fe, Fe_2_B, as well as Fe_3_P phases.

**Figure 2:**
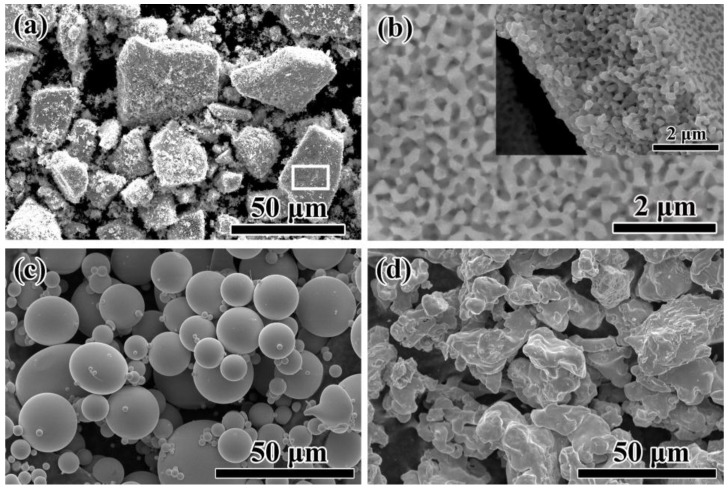
SEM image of NP powders (**a**); high magnification SEM image of NP powders (**b**) in the frame marked in (**a**); AM powders (**c**); and FE powders (**d**). The inset is the cross sectional image of dealloyed Fe_76_Si_9_B_10_P_5_ alloy.

**Figure 3:**
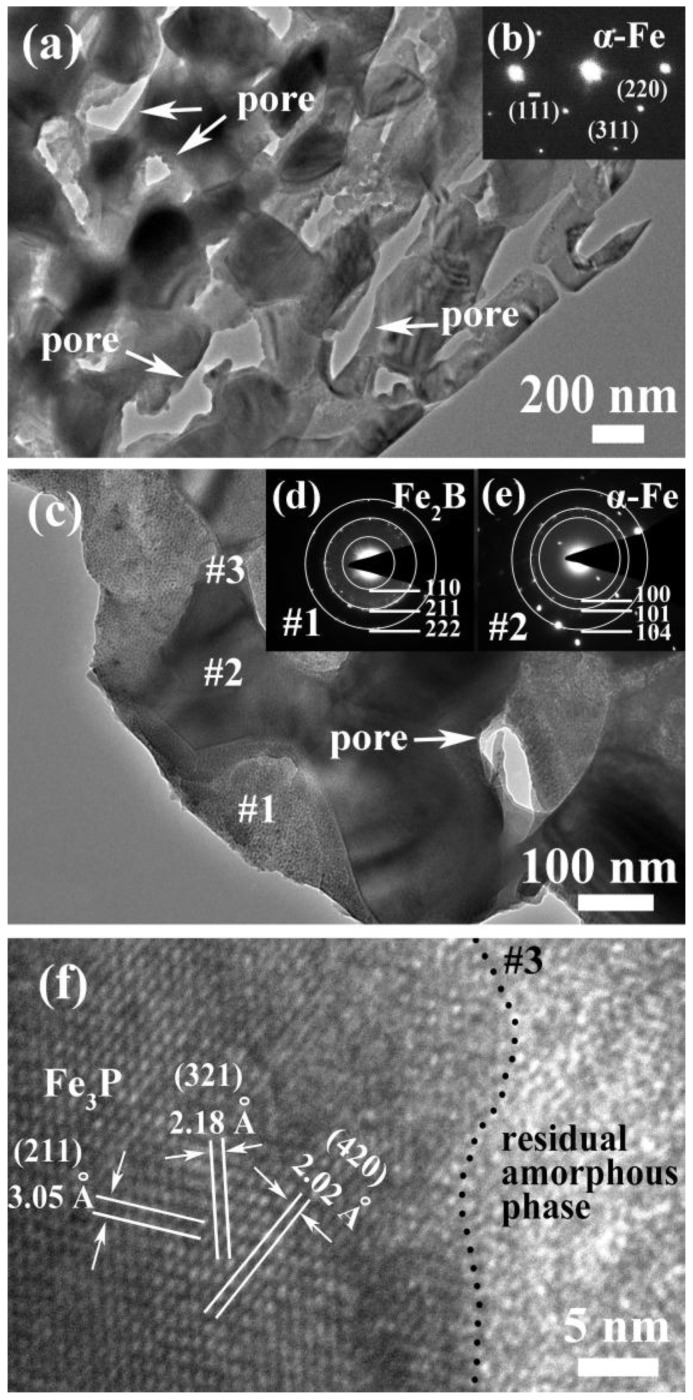
Low- and high-magnification bright field TEM images of NP powders (**a**,**c**); SADP patterns in a wide zone in (**b**); and Site 1# and 2# (**d**,**e**); and high-resolution TEM image (**f**) of dealloyed Fe_76_Si_9_B_10_P_5_ alloy at Site 3#.

The morphologies of NP powders, AM powders and FE powders are shown in Figure 2. The dealloyed ribbons were broken into small powders with the average size of about 20 μm as irregular shapes by milling (Figure 2a). As shown in high magnification image of Figure 2b, a homogeneously nanoporous structure with nanopores and continuous ligaments with a porosity of about 53% formed after dealloying in 0.05 M H_2_SO_4_ solution for 3.6 ks (Figure 2b). The cross-sectional SEM image of NP powders shows that inner part of the specimen is also in porous structure, meaning that the specimen is fully dealloyed (inset of Figure 2b). The AM powders prepared by gas atomization process are in a spherical shape with an average diameter of 18 μm (Figure 2c). Commercial Fe powders with a rough surface are in irregular shapes with a large particle size (about 42 μm) than AM powder (Figure 2d). The TEM images of dealloyed specimen show the microstructure with bi-continuous pores and ligament networks in Figure 3a. The selective area diffraction pattern (SADP) of NP powders in Figure 3b suggests the existence of α-Fe crystals based on the diffraction pattern from α-Fe (1–11), (220) and (311) crystal planes. Nanopores with a pore size of about 100 nm can be observed in the magnified TEM image in Figure 3c. The difference in the contrast might be attributed to the difference in the chemical composition. The SADPs in Figure 3d,e are conducted at Sites 1# and 2# in Figure 3c. Residual phases in gray contrast at Site 1# were identified as Fe_2_B with crystal planes of (110), (211) and (222) and α-Fe phases with diffraction indexed as (100), (101) and (104) at Site 2#. The high resolution TEM image in Figure 3f at Site 3# denotes that there exists small amount of residual amorphous phase after dealloying and Fe_3_P phases with crystal plane spacing of 3.05 Å, 2.18 Å and 2.02 Å. The results of TEM are consistent with the results of XRD patterns in Figure 1 which also demonstrates the complex existence of different phases in the NP powders. Although most of the residual phases are confirmed as crystalline α-Fe, Fe_2_B and Fe_3_P phases, small amount of amorphous phase coexists in the ligaments.

**Figure 4:**
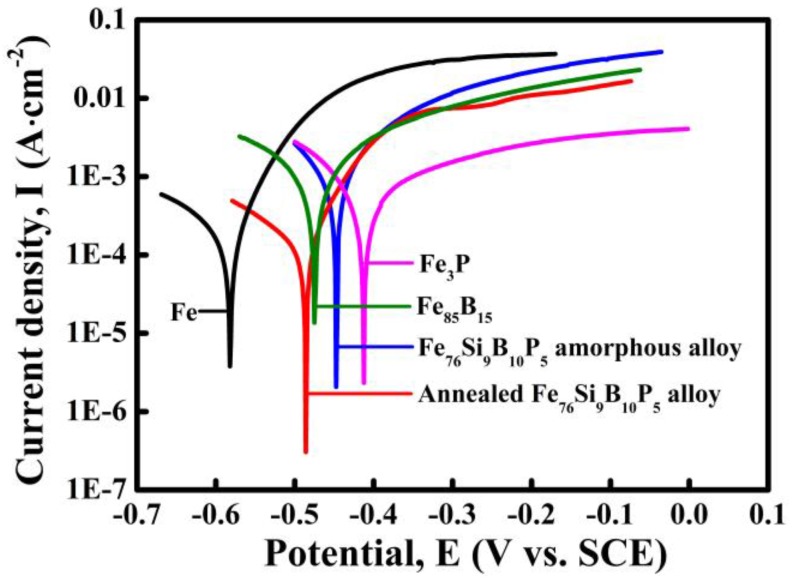
Polarization curves of the high-purity Fe plate, Fe_85_B_15_ alloy, Fe_3_P alloy, as-spun Fe_76_Si_9_B_10_P_5_ amorphous alloy and annealed Fe_76_Si_9_B_10_P_5_ alloy in 0.05 M H_2_SO_4_ solution.

The potentiodynamic polarization behavior of the high-purity Fe plate (α-Fe), Fe_85_B_15_ alloy, Fe_3_P alloy, as-spun Fe_76_Si_9_B_10_P_5_ amorphous alloy and annealed Fe_76_Si_9_B_10_P_5_ alloy was investigated in 0.05 M H_2_SO_4_ solution. As shown in Figure 4, all the tested alloys are actively dissolved in 0.05 M H_2_SO_4_ solution with different corrosion potentials. The corrosion potential of Fe plate is −0.58 V, which is much lower than those of Fe_85_B_15_ alloy (−0.47 V), Fe_3_P alloy (−0.41 V), as-spun Fe_76_Si_9_B_10_P_5_ amorphous alloy (−0.45 V) and annealed Fe_76_Si_9_B_10_P_5_ alloy (−0.48 V). It is concluded that high-purity Fe plate has the highest dissolution activity in 0.05 M H_2_SO_4_ solution. From the electrochemical aspects, high-purity Fe plate tends to start the preferential dissolution once Fe plate couples with Fe_85_B_15_ alloy, Fe_3_P alloy, as-spun Fe_76_Si_9_B_10_P_5_ amorphous alloy or annealed Fe_76_Si_9_B_10_P_5_ alloy. On the one hand, the negative shift of corrosion potential of annealed Fe_76_Si_9_B_10_P_5_ alloy is considered to result from the precipitation of higher electrochemical-active α-Fe nanocrystals during annealing. On the other hand, the decrease in the intensity of the main diffraction peak of α-Fe phase after dealloying in Figure 1 to some extent proves the preferential dissolution of the α-Fe phase in annealed alloy. The electrochemical stability of these tested alloys reflects the order of the preferential reactions in the dealloying process which might influence the degradation behavior of azo dye-containing industrial wastewater.

### 3.2. Enhanced Azo Dye Degradation Performance of NP Powders

**Figure 5:**
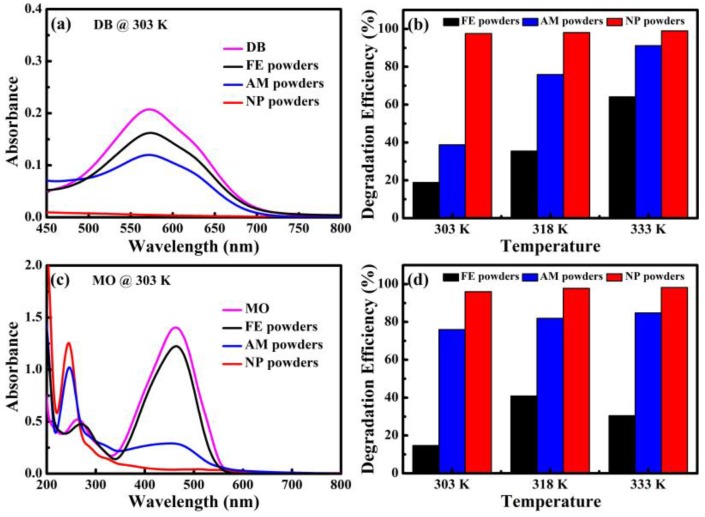
UV-Vis spectra (**a**,**c**); and degradation efficiencies (**b**,**d**) of: DB (**a**,**b**); and MO (**c**,**d**) degraded by NP powders, AM powders and FE powders at different temperatures.

The UV-Vis spectra and degradation efficiency of MO and DB degraded by NP powders, AM powders and FE powders are shown in Figure 5. Degradation efficiencies of azo dye by NP powders, AM powders and FE powders were calculated using Equation (1) [18]:

η = (A_0_ − A)/A_0_ × 100% (1)

where η is degradation efficiency, A_0_ is the original absorbance and A is the absorbance of degraded solutions. The corresponding degradation efficiencies of DB and MO solutions at different cases are listed in Table 1. Only one peak at 576 nm corresponding to the wavelength of green and yellow color could be observed in the UV-Vis spectra of DB solution in Figure 5a. The absorbance peak of the DB solution after reacting with NP powders is much lower than those of the two other kinds of powders within the same degradation time of 25 min. The degradation efficiencies of DB for NP powders are 97.6%, 98.1% and 99.0%, at 303 K, 318 K and 333 K, respectively. The degradation of DB solution until colorless for NP powders takes 8 min at 303 K. When the degradation is performed at higher temperature, the degradation of DB solution until colorless for NP powders decreases to 4 min and 3 min, at 318 K and 333 K, respectively. Meanwhile, degradation efficiencies of AM powders and FE powders are 38.8% and 18.9% at 303 K in Figure 5b. The degradation efficiencies of DB by AM powders and FE powders also increase with the increasing of the temperature. It is concluded that the NP powders degrade the DB solution much faster than AM powders and FE powders. In addition, two absorbance peaks in the UV-Vis spectra of MO solution degraded by three kinds of powders at 303 K are shown in Figure 5c. The broad peak at 463 nm is originated from –N=N– bonding of MO and a new absorbance peak at about 245 nm appearing after degradation reaction is considered to be reaction products after degradation because some intermediates forms during the process of degradation reaction [7]. The peak intensity at 463 nm is much weaker while the peaks at 245 nm become stronger after reacting with MO by NP powders compared with those by AM and FE powders, suggesting that NP powders exhibit much better degradation performance in the same condition. The degradation times till colorless of MO solution for NP powders are 60, 34 and 25 min at 303 K, 318 K and 333 K, respectively. The degradation efficiencies of NP powders are higher than 96% at all temperatures (Table 1). It is obvious that NP powders demonstrate higher degradation efficiency for MO compared with AM and FE powders. Similarly, the degradation efficiency of MO solution by NP powders is further improved with the increasing of the temperatures, accompanying with the degradation time decreasing significantly.

**Figure 6:**
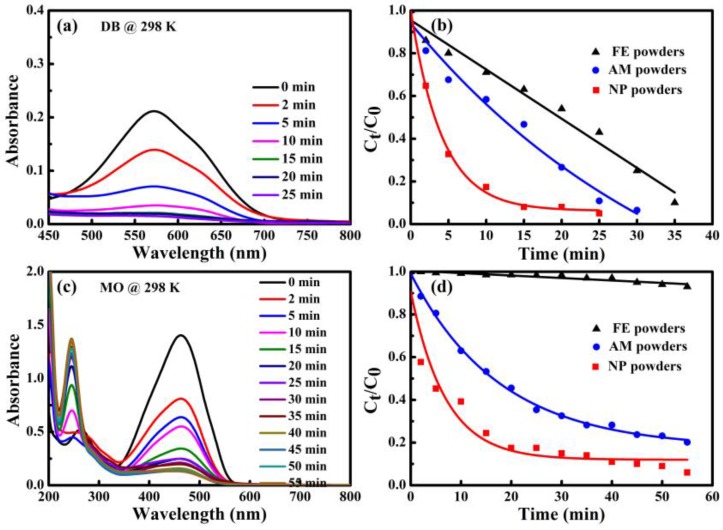
UV-Vis spectra of: DB (**a**); and MO (**c**) vs. time during the degradation process at 298 K by NP powders. The normalized concentration (*C*_t_/*C*_0_) vs. time of: DB (**b**); and MO (**d**) degraded by NP powders, AM powders and FE powders at 298 K.

**Figure 7:**
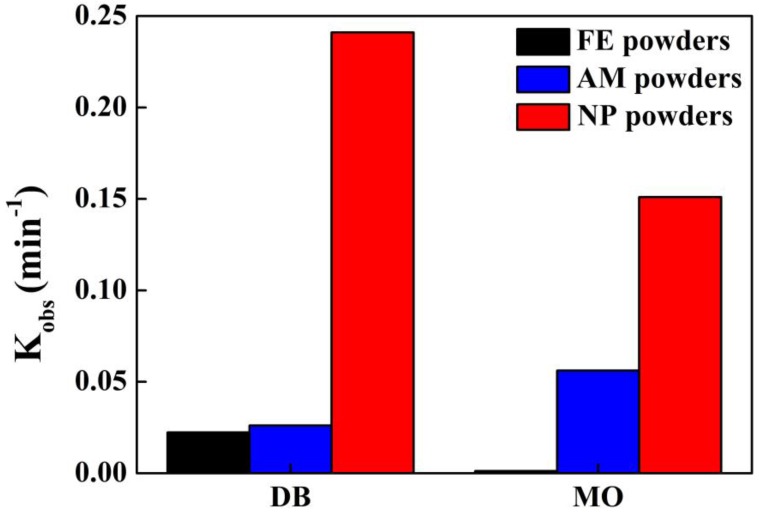
Reaction rate constant *k*_obs_ of DB and MO solutions degraded by NP powders, AM powders and FE powders.

The degradation behavior of DB and MO by three kinds of powders is investigated based on UV-Vis spectra taken at different degradation times at 298 K. For DB solution, as shown in Figure 6a, the absorbance peak at about 576 nm corresponding to color radical decayed with the evolution of time in the UV-vis spectra. The peaks almost totally disappear after degradation of 25 min. The normalized concentration *C*_t_/*C*_0_ variation of DB against time derived from UV-Vis spectra is shown in Figure 6b. Here, *C*_t_ and *C*_0_ are real-time and initial concentration of DB solution. It is found that *C*_t_/*C*_0_ decreases drastically in the case of degradation by NP powders, especially at the beginning of degradation. The value of *C*_t_/*C*_0_ is less than 0.2 after degradation for 10 min, implying that more than 80% of DB azo dye has been decomposed. Accordingly, about 40% and 25% of DB are degraded completely by AM powders and FE powders, respectively. In contrast, the UV-Vis spectra for MO have two peaks centered at 463 nm and 269 nm before degradation treatment in Figure 6c. As the degradation reactions undergo, the absorption peaks at 463 nm of the chromophore of the –N=N– bonding become lower, while the peaks at 269 nm shift to 245 nm and the peaks become stronger and stronger, suggesting that the azo –N=N– bonding has been broken, accompanying with the formation of some intermediates containing the benzene ring [7,19]. By using the absorption intensity at 463 nm, the normalized concentration change against time of MO was acquired (Figure 6d). The NP powders exhibits the much better degradation performance than AM powders and FE powders. Compared with Figure 6b, the plots of *C*_t_/*C*_0_ vs. time of FE powders in Figure 6d is different. The degradation of DB and MO solutions by both NP powders and AM powders follows the pseudo-first-order kinetic model (*C = C*_0_ exp (−*k*_obs_*t*), *R*^2^ > 0.95), and FE powders obeys the pseudo-zero-order kinetic model (*C* = *C*_0_(1 *− k*_obs_*t*)). All the calculated reaction rate constants (*k*_obs_) are presented in Figure 7 and Table 1. The *k*_obs_ value of the degradation of DB molecule by NP powders could be about 9 and 10 times higher than those of AM powders and FE powders, respectively. The *k*_obs_ value of the degradation of MO by NP powders is three times higher than AM powders and 131 times higher than FE powder. The MO azo dye (C_14_H_14_N_3_SO_3_Na) and DB (C_32_H_20_N_6_S_4_O_10_Na_4_) have molecular weights of 360 g mol^−1^ and 1029 g mol^−1^, respectively. DB azo dye is regarded as a representative large molecule and MO azo dye does as a medium molecule. FE powders show a better degradation ability for large molecules than medium molecules, while AM powders perform better on medium molecules. Among the three powders, the NP powders have the best degradation performance on both large molecules of DB and medium molecules of MO, which might be due to the diversity in the chemical composition of the nanoporous structure because of the coexistence of amorphous phase and crystalline α-Fe, Fe_2_B and Fe_3_P phases.

**Figure 8:**
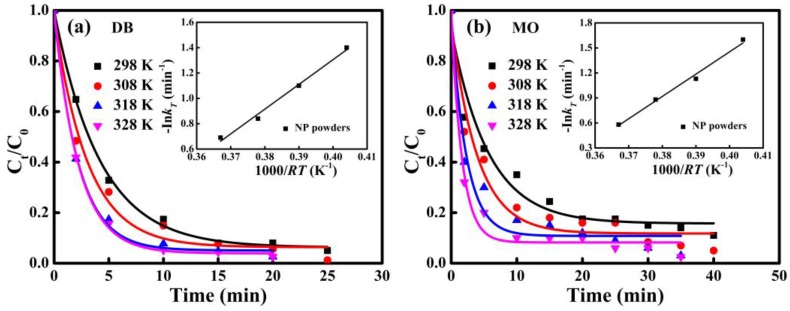
The normalized concentration (*C_t_*/*C*_0_) vs. time of: DB (**a**); and MO (**b**) by NP powders at 308 K, 318 K and 328 K. The Arrhenius plots of degradation of DB and MO by NP powders are inserted correspondingly.

The normalized concentration *C*_t_/*C*_0_ and the activation energy of the degradation reactions of DB and MO azo dyes by NP powders from 298 K to 328 K are shown in Figure 8. Reaction rate constants at different temperatures can be deduced by fitted data from the pseudo-first-order model. According to the Arrhenius equation, the activation energy can be derived from the change of temperature using Equation (2) [20,21]:

ln*k_T_* = −Δ*E/RT* + lnA (2)

where *k_T_* is the kinetic rate constant at different temperatures (*T*), Δ*E* is the activation energy (kJ mol^−1^), *R* is the gas constant and A is a constant. The plot of -ln*k_T_* versus 1000/*RT* for DB and MO solutions are inserted in Figure 8a,b. Good linear relationships have been obtained (*R*^2^ > 0.95), and the calculated activation energies for the degradation of DB and MO by NP powders are 19.5 kJ mol^−1^ and 26.8 kJ mol^−1^, which are much lower than those of commercial [7], demonstrating excellent degradation performance of NP powders.

### 3.3. Enhanced Degradation Mechanism of NP Powders

**Figure 9:**
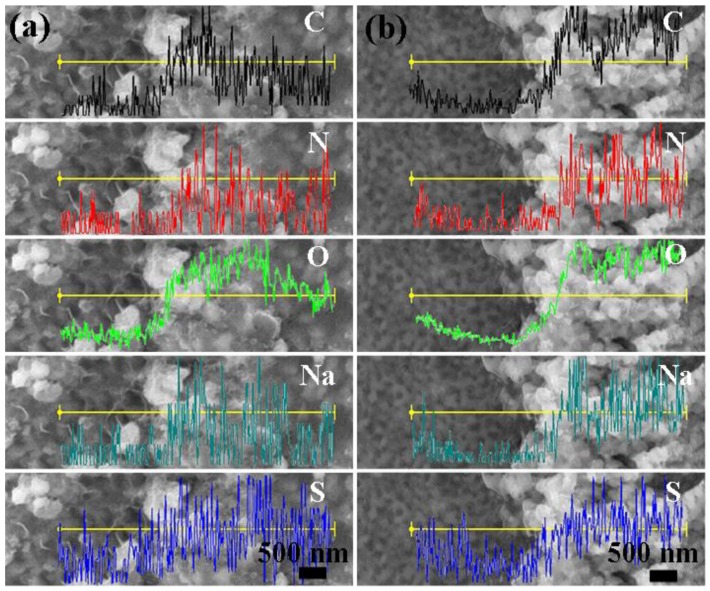
The EDS line scanning of the surface of NP powders after reacting with: DB solution (**a**); and MO solution (**b**).

To explore the degradation mechanism of NP powders, the surface morphology and elemental distribution of the powders after reaction with the azo dye solutions were analyzed. Figure 9 displays the surface morphology of NP powders after reacting with DB (Figure 9a) and MO (Figure 9b) solutions. Instead of fine and clear pores in the full scale, as shown in Figure 2b, some white particles and floccule-like products, which might be the decomposition products of azo dyes, cover the porous surface. The EDS line scanning results of the specimens reacted with azo dye solutions illustrate the variation of the composition along the yellow line. After reaction with azo dyes solutions, the amount of Fe is decreased from 86.4% to 15.4%, while small amounts of C, N, S and Na elements can be detected in the white products, which are constituent elements of azo dyes.

**Figure 10:**
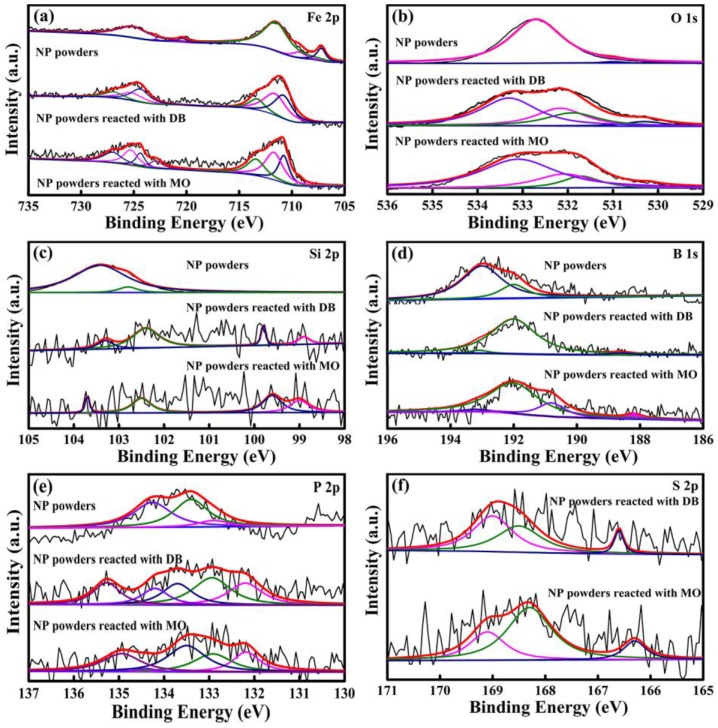
The XPS spectra of: Fe 2*p* (**a**); O 1*s* (**b**); Si 2*p* (**c**); B 1*s* (**d**); P 2*p* (**e**); and S 2*p* (**f**) of the surface products before and after degradation of DB and MO by NP powders.

Moreover, to obtain the detailed elemental information from the outermost atomic layers, XPS measurement was implemented to identify the surface chemical states of NP powders before and after reaction. The XPS spectra are summarized in Figure 10. Before reaction, all of the alloy elements (Fe, Si, B, P) as well as absorbed C and O elements are detected. Fe 2p spectrum in original NP powders is composed of a mixture of Fe^0^ (707.2 eV), Fe^2+^(709.3 eV) and Fe^3+^(711.5 eV), as displayed in Figure 10a. B*_x_*O*_y_* (B^3+^, 192.2 eV), B_2_O_3_ (B^3+^, 193.1 eV), Si^0^ (98.9 eV), SiO (Si^2+^, 102.3 eV) and SiO_2_ (Si^4+^, 103.8 eV) are also observed. In contrast, the concentrations of Si, B, and P elements are far lower than those in the original surface of NP powders after reaction, hinting that these metalloids are involved in the degradation reaction. The existence of Si^4+^/Si^2+^, B^3+^ and P^5+^ indicates that the metalloids are oxidized during degradation reaction. Fe 2*p* spectra in Figure 10 are mainly composed of Fe^3+^ from Fe_2_O_3_ (Fe^3+^, 710.8 eV), γ-FeOOH (Fe^3+^, 711.7 eV), and Fe_2_(SO_4_)_3_ (Fe^3+^, 713.4 eV) after reacting with azo dyes. It should be noted that small amount of S and Na elements (0.18–0.22 at %) rising from SO_4_^2+^/SO_3_^2+^ and Na^+1^ species are confirmed on the surface of NP powders after degradation with azo dyes, demonstrating the formation of Na_2_SO_4_ and Na_2_SO_3_ [7,22].

**Figure 11:**
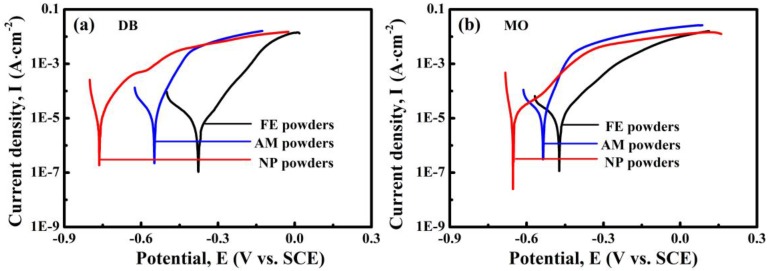
Polarization curves of NP powders, AM powders and FE powders in: DB solution (**a**); and MO solution (**b**).

The potentiodynamic polarization curves of NP powders, AM powders and FE powders in 50 mg/L DB and 20 mg/L MO solutions were investigated to discuss the chemical stability of the different powders, as shown in Figure 11. All the tested specimens are actively dissolved during the anodic polarization process. In both solutions, NP powders show much lower corrosion potential than those of AM powders and FE powders, hinting that NP powders exhibit higher electrochemical activity in both the azo dye solutions, accompanied with more active catalytic activity during the process of degradation.

### 3.4. Discussion

As mentioned above, Fe_76_Si_9_B_10_P_5_ amorphous alloy was crystallized to form α-Fe, Fe_2_B and Fe_3_P phases after annealing. During the process of dealloying in 0.05 M H_2_SO_4_ solution, a nanoporous structure formed because of the partially preferential dissolution of α-Fe phase. In our previous research [23,24], α-Fe grains in nanocrystalline Fe_83.3_Si_3_B_10_P_3_Cu_0.7_ and Fe_85.2_B_10–14_P_0–4_Cu_0.8_ alloys are preferentially dissolved in the form of micro-coupling cells between anodic α-Fe grains and cathodic residual amorphous phases. In this research, active dissolution of high-purity Fe plate, Fe_85_B_15_ alloy, Fe_3_P alloy, and as-spun Fe_76_Si_9_B_10_P_5_ amorphous alloy in Figure 4 to some extent reflects the active dissolution of α-Fe, Fe_2_B and Fe_3_P phases in the matrix of the annealed Fe_76_Si_9_B_10_P_5_ precursor alloys during dealloying. It is predictable that Fe_2_B phase in the annealed alloys exhibits a higher corrosion potential than that of Fe_85_B_15_ alloy because the ratio of B/Fe in Fe_2_B is higher than that in Fe_85_B_15_ alloy. Thus, α-Fe grains own the highest electrochemical activity among α-Fe, Fe_2_B, Fe_3_P and residual amorphous phases (confirmed by XRD and SADPs in Figure 1 and Figure 3) and undergo the preferential dissolution of α-Fe grains in 0.05 M H_2_SO_4_ solution. The selective dissolution behavior of α-Fe grains in annealed Fe_76_Si_9_B_10_P_5_ alloy is similar to the reported mechanism although the present reactions are more complicated due to the involvement of other Fe_2_B and Fe_3_P phases [23,24]. In other words, α-Fe grains dissolved quickly once exposed to H_2_SO_4_ solution, while the residual amorphous phases, Fe_2_B and Fe_3_P, with higher corrosion potentials, dissolve slowly, as shown in the results presented in Figure 3 and Figure 4.

On the other hand, the NP powders with a nanoporous structure exhibit higher degradation efficiency to azo dye solutions than the AM powders and FE powders. The enhanced degradation performance of NP powder may be elaborated by the following reasons. One reason is that the surface area of the NP powders with the formation nanoporous architecture increases significantly which are beneficial to the adsorption of dye molecules, supporting more reduction and oxidation sites for degradation reaction. The Fe element, as well as the metalloids (Si, B and P), in the ligaments of the nanoporous structure, acting as electron donors, will be oxidized when degradation reaction occurs. At the same time, the azo-dye molecular chains as acceptors are broken after the capture of electrons. Furthermore, based on the TEM image (Figure 3a) and cross-sectional SEM image (Figure 2b), the nanopores inside NP powders interconnected together which may form the fluid channels for azo dye solution, which further improve the degradation efficiency. From the view of electrochemical activity, it is also possible to explain why NP powders show higher degradation performance to azo dyes than AM powders and FE powders. The differences in the corrosion potential between FE powders and AM powders in DB and MO solutions in Figure 11 are 170 mV and 110 mV, respectively, which mainly result from the difference in the chemical composition and microstructure. The differences in the corrosion potential between AM powders and NP powders in DB and MO solution in Figure 11 are more than 290 mV and 110 mV, respectively, which are believed to originate from the difference in microstructure. The lower corrosion potentials of NP powders in azo dye solutions imply that NP powders exhibit higher electrochemical activity, accompanied with more active catalytic activity during the process of degradation with a lower activation energy. Another reason is that the coexistences of the residue amorphous phase in metastable state and metalloid elements such as Si, B and P after dealloying may enhance the catalytic activity of degradation reaction more by forming loose metalloid-containing intermediate products [8]. Here, the excellent catalytic activity of amorphous electrodes results from the abundance of the active sites induced by the special electronic structure [25]. Thus, the degradation process by NP powders is accelerated compared with AM powders and FE powders.

According to the above mentioned results, the degradation process of NP powders can be summarized as followings: MO or DB molecules are firstly absorbed to the nanoporous architecture, which are bicontinuous pore-and-ligament networks with large reactive surface area, then Fe elements were oxidized to Fe^3+^. Meanwhile, the cleavage of nitrogen to nitrogen double bonds (–N=N–), which are the most active bonds in azo dye molecules, leads to the decoloration of azo dyes. Whenazo dyes are degraded to intermediates, which are partially attached on the porous structure, the intermediates are subsequently further oxidized to small molecules, such as CO_2_, H_2_O and Na_2_SO_4_/Na_2_SO_3_ [22]. In addition, the coexistence of residue amorphous phase in metastable state and metalloids in porous ligament, which makes the surface more active by forming loose oxide layer in the process of degradation, may also play an important role to enhance the degradation activity [7,8].

## 4. Conclusions

The Fe-Si-B-P nanoporous architecture was obtained by dealloying of annealed Fe-Si-B-P amorphous alloys because of the preferentially dissolution of α-Fe grains as a consequence of its lower potential in the dealloying process. The NP powders have much better degradation efficiency as a result of their large surface area and porous structure compared to those of AM powders. The activation energies for the degradation of DB and MO by NP powders are estimated to be 19.5 kJ mol^−1^ and 26.8 kJ mol^−1^. High surface area of the nanoporous structure, the existence of metalloids, and residual amorphous phase with high catalytic activity are responsible for the enhanced degradation activity of the NP powders to azo dyes. The investigated Fe-Si-B-P nanoporous materials with high surface area and excellent catalytic activity to degrade azo dyes exhibit a high application potential in the field of the treatment of industrial wastewater in the future.

## Figures and Tables

**Table 1:** The degradation Efficiency (η) and the reaction rate constants (*k*_obs_) of DB and MO degraded by three kinds of powders at different temperatures.

Powders	Degradation Efficiency (%)						*K*_obs_ (min^−1^)	
	DB			MO			DB	MO
	303 K	318 K	333 K	303 K	318 K	333 K	298 K	
FE powders	18.9	35.5	64.1	14.7	40.9	30.5	0.0223	0.0012
AM powders	38.8	75.9	91.2	76.0	81.9	84.8	0.0261	0.0561
NP powders	97.6	98.1	99.0	96.0	97.8	98.2	0.2410	0.1510
